# Non-prescription dispensing of veterinary medicines for treating mastitis in dairy cattle among non-veterinary personnel in selected districts of Zambia

**DOI:** 10.3389/fvets.2025.1707434

**Published:** 2026-01-02

**Authors:** Amon Bwalya, Ethel M’kandawire, Alison Pyatt, Catrina Prince, Claire Gilbert, John Bwalya Muma, Steward Mudenda

**Affiliations:** 1 Department of Disease Control, School of Veterinary Medicine, University of Zambia, Lusaka, Zambia; 2 Veterinary Medicine Directorate (VMD-UK), Addlestone, United Kingdom; 3 Department of Pharmacy, School of Health Sciences, University of Zambia, Lusaka, Zambia

**Keywords:** antimicrobial resistance, dairy cattle, mastitis, dispensing, non-prescription sale veterinary medicines, veterinarian, veterinary paraprofessional, agrovet

## Abstract

**Objectives:**

Veterinary antibiotics are vital for managing bacterial infections in animals and preventing zoonotic transmission through animal-derived foods, direct contact, or the environment. However, their misuse and overuse contribute to the emergence and spread of antimicrobial resistance (AMR) across humans, animals, and ecosystems. This study assessed the dispensing practices of non-veterinary personnel regarding veterinary medicines used in dairy cattle in selected districts of Zambia. Specifically, it estimated the prevalence of non-prescription dispensing of antibiotics for treating mastitis and identified the most commonly dispensed antibiotic classes.

**Methods:**

A cross-sectional study was conducted between September 2024 and June 2025 among 220 veterinary medicine dispensers in Lusaka, Chisamba, Kafue, Chongwe, Monze, and Choma districts. Data were collected using a simulated farmer (mystery shopper) approach to document veterinary antibiotics and other medicines dispensed for treating mastitis in dairy cattle at retail pharmacies and agro-veterinary shops.

**Results:**

Of the 220 outlets, 74 (33.6%) were agro-veterinary shops and 146 (66.3%) retail pharmacies. Overall, 126 outlets did not stock veterinary medicines for mastitis. Among the 94 that did, 85 (90.4%) dispensed antibiotics and 9 (9.6%) dispensed other medicines without a prescription. Of the 134 veterinary medicines dispensed, 74.6% (*n* = 100) were antibiotics, followed by anti-inflammatories (11.2%), supplements (7.5%), hormones (4.5%), and antiparasitics (2.2%). Common antibiotic classes included penicillins, aminoglycosides, tetracyclines, and cephalosporins. Only 53.2% of attendants asked about symptoms, and among them, 54.7% provided a tentative diagnosis. Few inquired about prior treatment (8.5%), advised on disease progression (19.1%), or referred the simulated farmer to a veterinarian (12.3%). While 74.5% communicated the correct route of administration, only 63.8% mentioned dosage frequency, and 58.5% specified treatment duration.

**Conclusion:**

Non-prescription dispensing of veterinary antibiotics for mastitis by non-veterinary personnel is widespread, posing significant AMR risks to animal, human, and environmental health. Strengthened regulatory oversight, antimicrobial stewardship training, and collaboration between veterinarians and drug outlet owners are essential to promote responsible antibiotic use and curb AMR.

## 1 Introduction

Veterinary medicines are essential for the maintenance of livestock health and productivity, with responsible prescribing practices playing a critical role in safeguarding both animal welfare and food safety (1–3). Accurate prescription and administration are key to preventing the spread of infectious diseases and mitigating the growing threat of antimicrobial resistance (AMR) (4). However, in many low- and middle-income countries (LMICs), concerns have been raised for over a decade regarding the involvement of non-veterinary personnel in the prescribing and administration of veterinary medicines (5, 6). This practice has led to inappropriate use; inadequate professional training and regulatory oversight are indicated as causative factors of note (7, 8).

Veterinarians and in some countries, veterinary para-professionals (VPPs) are authorized to prescribe antibiotics, with expectations that such prescriptions are guided by principles of antimicrobial stewardship (AMS), accurate diagnosis, and appropriated monitoring of treatment outcomes (6, 9). AMS comprises coordinated interventions aimed at promoting the appropriate use of antimicrobials, improving treatment outcomes, and minimizing unnecessary costs (10–12). Central to AMS is the accurate diagnosis and rational prescribing of medicines by trained veterinary professionals (10, 11). However, in many LMICs including Zambia, VPPs are not legally permitted to prescribe antibiotics, reflecting variations in national regulatory frameworks (13–15). According to Zambia’s Veterinary and Para-Veterinary Professions Act No. 45 of 2010 and the Medicines and Allied Substances Act No. 3 of 2013, antibiotics are classified as prescription-only medicines (POMs) that may be dispensed solely on the written authority of a registered veterinary surgeon (16, 17). Despite established protocols and regulatory guidelines, the non-prescription sale and use of veterinary antibiotics remain common in the many Sub-Saharan African countries (SSAC) including Zambia (18, 19).

The increasing global demand for dairy milk products has accelerated the use of veterinary medicines, particularly antimicrobials in LMICs (20, 21). This demand often drives intensive farming systems where antimicrobials are routinely used to prevent disease and promote growth, especially in the absence of robust veterinary oversight (22, 23). Approximately 73% of all antimicrobials sold globally are administered to food-producing animals, a practice that is especially prevalent in intensive livestock systems to prevent diseases in crowded or unsanitary conditions (24, 25). This widespread use of antibiotics creates selective pressure that facilitates the emergence and spread of antimicrobial-resistant bacteria within animal populations. Resistant pathogens can then be transmitted to humans through direct contact, consumption of animal products, or environmental pathways such as water runoff and manure application (21, 26). The persistence of AMR in these settings reduces the efficacy of both human and veterinary antimicrobial treatments, posing a serious global health threat (23). AMR is now widely recognized as one of the leading public health challenges of the 21st century (27), with substantial mortality attributed to antibiotic-resistant infections (28, 29). In LMICs, the non-prescription sale of antibiotics is common, particularly through agro-veterinary outlets, where access to veterinary professionals is limited (30). For example, in Ethiopia, studies have reported the non-prescription sale and dispensing of antibiotics and anthelmintics, contributing to the misuse of these products and worsening AMR (31). The proliferation of substandard and falsified medicines, driven by cost and ease of access, further complicates the situation (32). The use of these medicines exacerbates production challenges faced by small-scale livestock farmers across Sub-Saharan Africa (SSA), heightens the risk of AMR development, and may further compound public health risks associated with the consumption of animal-derived food products (33).

Non-veterinary supply routes play a crucial role in the distribution of veterinary medicines, particularly in rural and peri-urban areas where access to qualified veterinarians is constrained (7). These supply routes include formal retail outlets, such as registered agro-veterinary shops and pharmacies, as well as informal sources, including general stores and unregistered vendors, many of which are operated by individuals lacking formal veterinary qualifications (18, 34). It is estimated that over 70% of veterinary medicines are sourced through these outlets, primarily due to their affordability, geographic accessibility, and the absence of consultation fees (35, 36). For smallholder farmers, especially those in remote locations, these channels represent the most practical and dependable means of obtaining livestock medications, partly compensating for under-resourced public veterinary services and frequent stockouts of essential drugs (7, 37).

Despite their accessibility, the widespread reliance on non-veterinary supply routes raises serious concerns regarding the inappropriate use of veterinary medicines, circulation of counterfeit products, and the escalating risk of AMR (34, 38). These challenges underscore the importance of understanding and addressing dispensing practices within agro-veterinary shops and retail pharmacies, particularly for common livestock diseases such as mastitis in dairy cattle (6). Mastitis is one of the most prevalent and economically important diseases of dairy cattle worldwide, and it has been reported as a major cause of reduced milk yield, poor milk quality, and culling of dairy animals in Zambia and other sub-Saharan African countries (39, 40). The condition is also a leading driver of veterinary antimicrobial use, with antibiotics such as penicillin–streptomycin combinations, oxytetracycline, and intramammary formulations frequently prescribed or dispensed for its treatment (41). Consequently, mastitis provides an ideal study to evaluate the dynamics of dispensing practices, given its high incidence, significant economic impact, and role in shaping antimicrobial use patterns in the dairy sector (42–45). Addressing these challenges requires not only an understanding of the disease burden but also a systems-level response to strengthen the veterinary medicines supply chain (35). This calls for improved regulatory capacity, good access to licensed veterinary outlets, affordable quality-assured medical products, and the enforcement of strict manufacturing and trade policies to reduce reliance on counterfeit veterinary medicines (33, 46, 47).

In Zambia, research on the non-prescription sale of veterinary medicines by non-veterinary personnel remains limited, with a small number of existing studies primarily focused on poultry production (48, 49). Recent evidence indicated that antibiotics such as oxytetracycline and amoxicillin are frequently dispensed without a prescription by unqualified personnel operating in agro-veterinary outlets (50). The situation highlights the urgent need for enhanced AMS in veterinary practice (48).

Despite growing global concern over AMR, comprehensive data on dispensing practices in the dairy sector, particularly involving non-veterinary personnel, remain scarce in Zambia. This study assessed the dispensing practices of veterinary medicines among non-veterinary personnel in Lusaka, Chisamba, Kafue, Chongwe, Monze, and Choma districts of Zambia, providing much-needed insight into an under-researched area. Given the similarities in veterinary medicine access, regulation, and livestock production systems across many low- and middle-income countries in the region, these findings may have broader relevance and could be extrapolated to neighboring countries facing similar challenges.

## 2 Materials and methods

### 2.1 Study design, settings, and population

**Figure 1:**
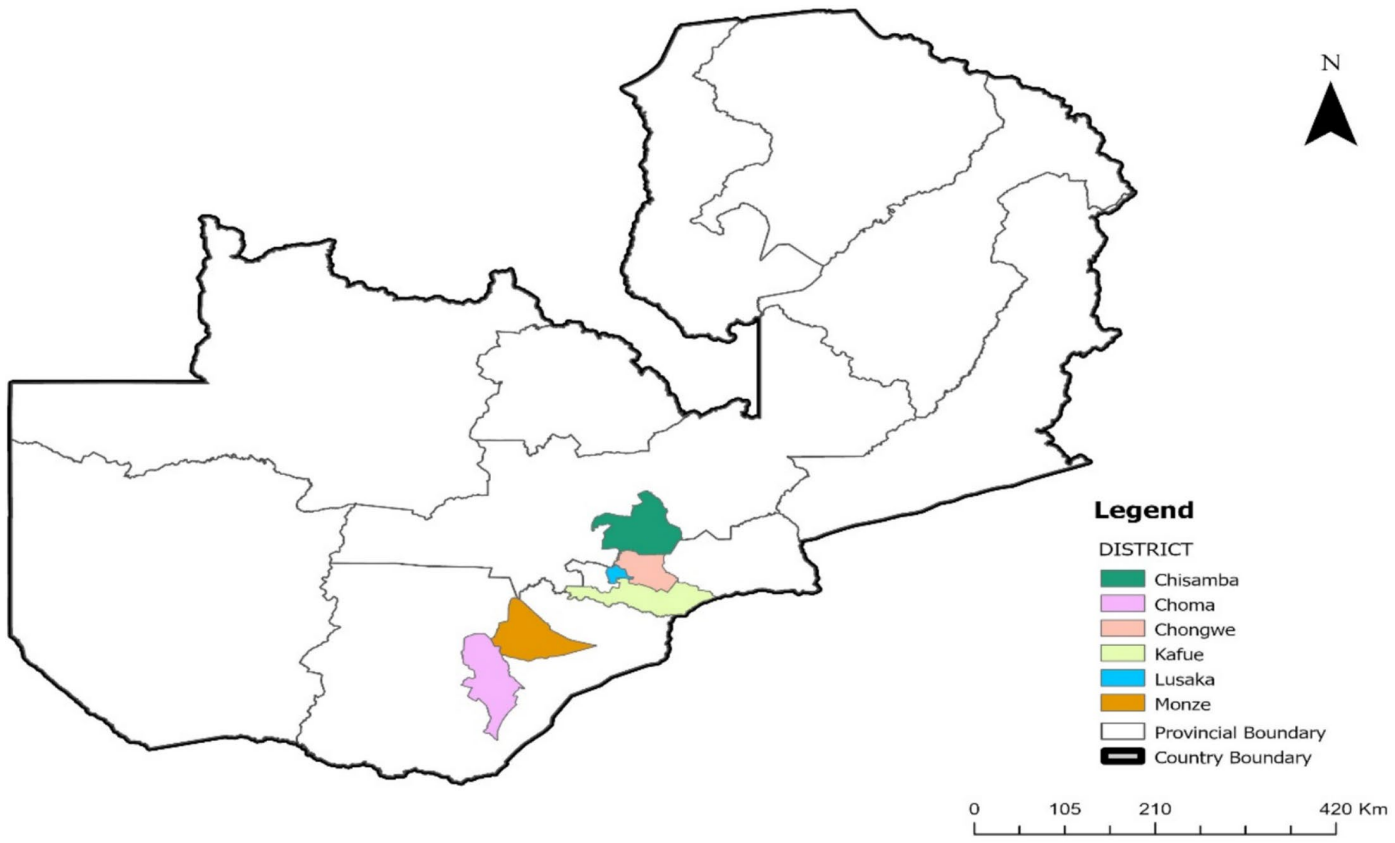
A map showing data collection site in selected districts of Zambia.

A cross-sectional study was conducted from September, 2024 to June, 2025 to assess the dispensing practices of veterinary medicines for treating mastitis in dairy cows among non-veterinary personnel in Lusaka, Chilanga, Chongwe, Kafue, Monze and Choma districts of Zambia. These sites were selected due to a significant presence of dairy farmers, pharmacy and agro-veterinary retail outlets, alongside limited access to qualified veterinarians for farmers seeking veterinary advice. Participants were purposively sampled from pharmacies and agro-veterinary shops registered with the Zambia Medicines Regulatory Authority (ZAMRA) (51). To be eligible, participants had to work in agro-veterinary or pharmacy settings, engage in the sale or dispensing of veterinary medicines, and lack formal veterinary qualifications (Figure 1).

### 2.2 Sample size estimation and sampling techniques

The target population consisted of personnel from 400 retail pharmacies and 50 agro-veterinary shops registered by ZAMRA, totaling 450 premises. Sample size was calculated using Yamane’s (1967) formula (52) given as *n* = N/(1 + N(e)^2^), where n = Sample size, N = Total population size (450 premises), and e = Level of precision (margin of error), being 0.05 at 95% confidence level. This yielded a sample size of 203 participants from the premises, broken down into approximately 25 agro-veterinary shops and 178 retail pharmacies. These were proportionally allocated to each of the six study districts based on the number of registered outlets in the district’s sampling frame. This ensured that districts with a higher number of premises contributed more to the sample than those with fewer outlets. However, during data collection, oversampling of pharmacies was deliberate to ensure adequate subgroup representation for comparative analysis, as the number of agrovets was limited in some districts.

### 2.3 Data collection

The data were collected using a mystery shopper approach tool adopted from a simulated farmer (SF) survey study (53). This approach allows the researcher to collect data without alarming the prescriber and other AMR studies have used this kind of tool (54). In this study, two SFs were trained by the principal supervisor (Steward Mudenda) to conduct visits to pharmacies and agro-veterinary shops, posing as dairy farmers with concerns over their cattle’s health. The trained SFs were selected based on prior experience in livestock management and communication skills. They underwent a two-day standardization and calibration training session on case presentation and structured observation methods. Inter-observer agreement was pre-tested and achieved >90% consistency. The two SFs would enter the pharmacy or agro-veterinary shop and introduce themselves as dairy cattle farmers and that they were worried that some of their animals are possibly not feeling too well as their milk production has gone down and the udder look somehow swollen and hence requesting the attendants to recommend and dispense some medicines that can be used to treat the possible infection. Based on the medicines dispensed by the attendant, the SF would write down the sex of the attendant, names of medicines dispensed after exiting the shop premises taking into consideration if the attendant asked any further questions regarding the history and other possible clinical symptoms noticed by the farmer. Further information was recorded regarding the frequency of dosing of any medicine dispensed, duration, and route of administration of the dispensed medicines. Recall bias was minimized by completing data forms immediately after each visit (Data collection tool as shown in Appendix 1).

### 2.4 Data analysis

Data collected from all non-veterinary personnel were entered into Microsoft Excel for initial cleaning before being transferred to Statistical Package for Social Sciences (SPSS) version 22.0 for detailed statistical analysis. Descriptive statistics including frequencies, percentages and means were used to summarize categorical and continuous variables. Chi-square tests or where necessary, Fishers Exact test were applied to compare categorical outcomes between pharmacies and agrovet outlets. Statistical significance was conducted at a 95% confidence level with a *p* < 0.05.

### 2.5 Ethical approval

Ethical approval was obtained from the University of Zambia Biomedical Sciences Research Ethics Committee (UNZABREC) in Lusaka, Zambia with reference number of 5,511–2024. The authority to conduct research was granted by National Health Research authority (NHRA) in Lusaka, Zambia with the reference number NHRA-1520/29/08/2024. Compliance with ethical standards and guidelines for research involving human subjects were respected and information collected for the study was restricted to the investigators and did not pose any risk to any participant. Furthermore, only codes and no names of community pharmacies or agro-veterinary shops were used during data collection and analysis to maintain anonymity and confidentiality.

## 3 Results

### 3.1 Characteristics of surveyed premises

In this study, data were collected from 220 premises across six districts, consisting of 74 (33.6%) agro-veterinary shops and 146 (66.4%) retail pharmacies. The majority of the premises were located in Lusaka (61.8%), followed by Chongwe, Kafue, Choma, Chisamba, and Monze. Retail pharmacies outnumbered agrovets in most districts, except Chisamba, where agrovets were more prevalent (Table 1).

**Table 1:** Distribution of agro-veterinary and retail pharmacy premises by district (*N* = 220).

District	Total premises (*n*)	Type of premise			
		Agro-veterinary (*n*)	Agro-veterinary %	Retail pharmacy (*n*)	Retail pharmacy %
Chisamba	15	10	66.7	5	33.3
Choma	19	5	26.3	14	73.7
Chongwe	20	8	40.0	12	60.0
Kafue	19	8	42.1	11	57.9
Lusaka	136	39	28.7	97	71.3
Monze	11	4	36.4	7	63.6
Total	220	74	33.6	146	66.4

### 3.2 Prevalence of antibiotic sale without prescriptions

Out of 220 agrovet and retail pharmacy premises surveyed, 94 (42.7%; 95% CI: 36.4–49.3) stocked and dispensed veterinary medicines, while 126 (57.3%) did not dispense any medications (Table 2). Among the 94 dispensers, 85 (90.4%) dispensed antibiotics without requiring a formal prescription (Table 3). Antibiotic sales were significantly higher in agro-veterinary shops (54.1%) compared with retail pharmacies (30.8%), χ^2^ = 10.8, *p* = 0.001 (Table 2).

**Table 2:** Summary of veterinary medicine dispensing practices and communication by attendants (*N* = 220).

Valuable	Frequency	Proportion (%)	Lower confidence interval (95% C.I)	Upper confidence interval (95% C.I)	Agrovet n/*N* (%)	Pharmacy *n*/*N* (%)	*p*-value
Dispensed any veterinary medicine	94	42.7	36.4	49.3	46/74 (62.2%)	48/146 (32.9%)	<0.001
Dispensed veterinary antibiotics	85	38.6	32.5	45.1	40/74 (54.1%)	45/146 (30.8%)	0.001
Quantity of antibiotics dispensed	100/134	74.6	66.5	81.2	51/74 (69.8%)	49/145 (33.6%)	<0.001
Asked the attendant about symptoms	117	53.2	46.6	59.7	47/74 (63.5%)	70/146 (47.9%)	0.041
Told the attendant the possible disease	64	29.1	23.5	35.4	34/74 (45.9%)	30/146 (20.5%)	<0.001
Attendant that asked about the previous medication	8	3.6	1.9	7.0	6/74 (8.1%)	2/146 (1.4%)	0.011
Attendant ask that asked about signs of infection to look for	18	8.2	5.2	12.7	9/74 (12.2%)	9/146 (6.2%)	0.162
Attendant that referred to a qualified veterinarian	27	12.3	8.6	17.3	11/74 (14.9%)	16/146 (11.0%)	0.137
Attendant that communicated the dose frequency	60	27.3	21.8	33.6	31/74 (41.9%)	29/146 (19.9%)	0.001
Attendant that communicated the correct duration of treatment	55	25	19.7	31.1	24/74 (32.4%)	31/146 (21.2%)	0.047
Attendant that communicated the correct mode of administration	70	31.8	26.0	38.2	30/74 (40.5%)	40/146 (27.4)	0.047

**Table 3:** Proportions of antibiotics dispensed without a prescription from the premises that dispensed veterinary medicines (*n* = 94).

Premise type	Veterinary medicine dispensers (denominator) (*n*)	Veterinary antibiotic dispensers (*n*)	Did not dispense antibiotics (*n*)	Proportions (%)	CI (95%)	*p*-value
Agro-veterinary shop	46	40	6	42.55	74.33–93.88	0.263
Retail pharmacy	48	45	3	47.87	83.16–97.85	0.263
Total	94	85	9	90.42	82.80–94.88	

### 3.3 Dispensing and diagnosis practices

The veterinary medicines were dispensed based on verbal complaints presented by the SF, with limited or no diagnostic questioning. Overall, 53.2% (117/220) of attendants asked about presenting symptoms. A significantly higher proportion of agro-veterinary shop attendants inquired about symptoms (63.5%) compared with pharmacy attendants (47.9%) (χ^2^ = 4.17, *p* = 0.041). Regarding diagnostic interpretation, 29.1% (64/220) of attendants offered a tentative diagnosis. Agrovet attendants provided tentative diagnoses more frequently (45.9%) than pharmacy attendants (20.5%) (χ^2^ = 15.10, *p* < 0.001). Key diagnostic questions were rarely asked. Only 3.6% (8/220) asked about prior treatment. Although overall proportions were low, agrovets (8.1%) were significantly more likely than pharmacies (1.4%) to inquire about earlier medication (χ^2^ = 6.46, *p* = 0.011). Inquiry about signs of disease progression was reported in 8.2% (18/220) of interactions, with no statistically significant difference between agrovets (12.2%) and pharmacies (6.2%) (χ^2^ = 1.96, *p* = 0.162). Referral to a licensed veterinarian occurred in 12.3% (27/220) of cases, with no significant variation between agrovets (14.9%) and pharmacies (11.0%) (χ^2^ = 2.21, *p* = 0.137).

Communication of treatment instructions was suboptimal across both premise types. Correct dosage was communicated in 27.3% (60/220) of visits, significantly more often in agrovets (41.9%) than pharmacies (19.9%) (χ^2^ = 11.53, *p* = 0.001). Similarly, communication of correct treatment duration was provided by 25.0% (55/220) of attendants, again higher in agrovets (32.4%) than pharmacies (21.2%) (χ^2^ = 3.97, *p* = 0.047). Appropriate mode of administration was explained in 31.8% (70/220) of interactions, with agrovets (40.5%) outperforming pharmacies (27.4%) (χ^2^ = 3.95, *p* = 0.047). Overall, agro-veterinary shops consistently demonstrated better diagnostic inquiry and communication practices than retail pharmacies, although performance across both groups remained suboptimal (Table 2).

### 3.4 Dispensing patterns of veterinary antibiotics by premise type

Of the 100 antibiotics dispensed without a formal prescription, 42.69% were dispensed by agrovet attendants and 47.87% by retail pharmacy attendants. Although retail pharmacies dispensed a slightly higher volume of antibiotics, the difference was not statistically significant (χ^2^ = 1.25, *p* = 0.263) (Table 3).

### 3.5 Types and classes of antibiotics dispensed

In this study, a total of 134 veterinary medicines were dispensed, of which 100 (74.6%) were antibiotics. The most commonly dispensed injectable antibiotics for treating mastitis were benzathine procaine penicillin and hydrostreptomycin sulfate (Penstrep) (30%) and procaine penicillin G and benzathine penicillin G (Megapen) (17%), followed by oxytetracycline (8%). For intramammary use, cefalexin monohydrate and kanamycin monosulphate (Terrexine) (19%) and Ampicillin and cloxacillin (Meltjet) (8%) were the most frequently dispensed antibiotics followed by tetracycline/neomycin/bacitracin/prednisolone (Mastijet Forte) (5%). Alarmingly, highest priority critically important antimicrobials (CIAs) such as ceftiofur (a third-generation cephalosporin) and fluoroquinolones (enrofloxacin and marbofloxacin), which are part of the WHO’s **“**Reserve**”** group under the AWaRe classification, were also dispensed (55) (Table 4).

**Table 4:** Most commonly dispensed antibiotics for mastitis treatment in community pharmacies and agrovet shops in selected districts of Zambia.

Name of product	Type of product	Quantity *n* (%)
Penstrep (Benzathine procaine penicillin and hydrostreptomycin sulfate)	Penicillin/aminoglycoside	30 (30%)
Terrexine (Cefalexin monohydrate and kanamycin monosulphate)	Cephalosporin/aminoglycoside	19 (19%)
Megapen (Benzathine procaine penicillin)	Penicillin	17 (17%)
Meltjet (Ampicillin and cloxacillin)	Penicillin	8 (8%)
Oxytetracycline	Tetracycline	8 (8%)
Mastijet Forte (Tetracycline/neomycin/bacitracin/prednisolone)	Tetracycline/aminoglycoside/glucocorticoid	5 (5%)
Gentamicin	Aminoglycoside	3 (3%)
Sulfatrim (Sulfamethoxazole and trimethoprim)	Potentiated sulphonamide	3 (3%)
Amoxicillin	Penicillin	2 (2%)
Marbofloxacin	Fluoroquinolone (Watch/Reserve)	2 (2%)
Florfenicol	Chloramphenicol	1 (1%)
Enrofloxacin	Fluoroquinolone (Watch/Reserve)	1 (1%)
Ceftiofur	Cephalosporin (Reserve – critically important)	1 (1%)

## 4 Discussion

This study found a widespread and concerning practice of non-prescription veterinary medicine sales in Zambia’s dairy sector. Among the 220 agrovet and pharmacy premises visited, 94 (42.7%) stocked veterinary medicines, and all 94 (100%) dispensed these medicines without a prescription. Notably, antibiotics alone accounted for 90.4% of all veterinary medicines dispensed. According to Zambia’s Veterinary and Para-Veterinary Professions Act No. 45 of 2010 and the Medicines and Allied Substances Act No. 3 of 2013, antibiotics are classified as prescription-only medicines (POMs) that may be dispensed solely on the written authority of a registered veterinary surgeon (16, 17). These findings are consistent with similar studies from other livestock sectors in Zambia. For instance, Mudenda et al. (2024) (48) reported 100% access to antibiotics without prescriptions in the poultry sector, underscoring a systemic regulatory failure. The practice of dispensing antibiotics without veterinary oversight is a recognized issue across many low- and middle-income countries (LMICs). Studies from Tanzania (94%) (30), Eritrea (89.2%), Ethiopia (87.9%), and Nigeria (86.5%) (56) have reported similar levels of unauthorized access. In Uganda, Samuel et al. (2023) also documented the non-prescription sale of antibiotics through agro-veterinary outlets and pharmacies (57). This trend has been attributed to weak regulatory enforcement, inadequate professional oversight, and limited awareness among sellers (4, 4). The implications are profound, as improper antibiotic access and use directly contribute to the development of AMR within the One Health framework (9, 10, 58). Although both agrovets and pharmacies participated in non-prescription sales, antibiotics were significantly more likely to be dispensed by agro-veterinary shops (54.1%) than by pharmacies (30.8%) (χ^2^ = 10.22, *p* = 0.001). This indicates that agrovets, often located closer to farming communities, are key nodes in non-prescription dispensing of antibiotic. However, the overall volume of antibiotics dispensed did not differ significantly between agrovets and pharmacies (χ^2^ = 1.25, *p* = 0.263), suggesting a comparable contribution to unregulated antibiotic access.

A total of 134 veterinary medicines were dispensed during this study, with antibiotics constituting the majority. The most commonly dispensed injectable antibiotics for treating mastitis were benzathine procaine penicillin and hydrostreptomycin sulfate (Penstrep) (30%), procaine penicillin G and benzathine penicillin G (Megapen) (17%), followed by oxytetracycline (8%). For intramammary use, cefalexin monohydrate and kanamycin monosulphate (Terrexine) (19%) were the most frequently dispensed antibiotics followed by ampicillin and cloxacillin (Meltjet) (8%) and then tetracycline/neomycin/bacitracin/prednisolone (Mastijet Forte) (5%). The most frequently dispensed antibiotic classes included penicillins, aminoglycosides, sulphonamides, fluoroquinolones, tetracyclines, cephalosporins, and chloramphenicol. These findings align with similar research in northern Tanzania and Ethiopia, where oxytetracycline and penicillin/streptomycin were the most dispensed antibiotics without prescription (31, 59). The classification of these antibiotics under the World Health Organization (WHO) Access, Watch, and Reserve (AWaRe) framework provides critical insight into their significance (55). While some antibiotics such as oxytetracycline and penicillin fall under the Access group, others like gentamicin, enrofloxacin, ceftiofur, and marbofloxacin belong to the Watch group; indicating a higher potential for resistance development (60–63). Notably, the widespread use of Watch group antibiotics, especially in non-prescription contexts, deviates from the initial WHO recommendation that at least 60% of all prescribed antibiotics should come from the Access group (64). Additionally, several antibiotics dispensed in this study including gentamicin and beta-lactams, are classified as critically important for human medicine (63, 65, 66). The frequent use of these antibiotics in livestock without veterinary supervision poses a significant risk to public health (67).

Dispensing and diagnostic practices were equally inadequate. Although overall clinical engagement was poor, significant differences were observed between the two premise types. For instance, agro-veterinary attendants asked about presenting symptoms more frequently (63.5%) than pharmacy attendants (47.9%) (χ^2^ = 4.17, *p* = 0.041). Similarly, agrovets offered tentative diagnoses more often (45.9%) than pharmacies (20.5%) (χ^2^ = 15.10, *p* < 0.001) and were more likely to inquire about prior treatments (8.1% vs. 1.4%, χ^2^ = 6.46, *p* = 0.011). However, inquiries about disease progression remained low across both groups. Referral to a qualified veterinarian was rare overall (12.3%) and did not significantly differ between agrovets (14.9%) and pharmacies (11.0%) (χ^2^ = 2.21, *p* = 0.137). Communication of treatment instructions was also suboptimal. Agrovets consistently outperformed pharmacies, explaining correct dosage (41.9% vs. 19.9%, χ^2^ = 11.53, *p* = 0.001), treatment duration (32.4% vs. 21.2%, χ^2^ = 3.97, *p* = 0.047), and mode of administration (40.5% vs. 27.4%, χ^2^ = 3.95, *p* = 0.047). Nonetheless, overall compliance with recommended dispensing and communication standards was low. These findings are similar though slightly fall short to the study previously conducted in Zambia (48), suggesting significant shortfalls in safe prescribing practices. Similar deficiencies in veterinary service quality have been reported in Ghana and Kenya, where farmers are often misinformed or not adequately supported (68, 69). In Kenya, a study observed that Maasai pastoralists frequently faced drug misuse risks due to poor advice and under-dosing (70). These findings reflect an urgent need to enhance training, regulation, and monitoring of agro-veterinary and pharmacy attendants who play a frontline role in animal healthcare so as to reduce the risk of AMR transmission and treatment failure in livestock.

### 4.1 Limitations of the study

While the mystery shopper approach provided reliable insights into actual dispensing practices, it relied on simulated scenarios rather than real clinical cases. This may not have fully captured the complexities of farmer–attendant interactions during genuine treatment consultations. The study was conducted in six districts of Zambia with high dairy activity. Although these districts provide valuable insights, the findings may not be generalizable to all regions of Zambia, especially areas with different livestock production systems or access to veterinary services.

The study concentrated on mastitis in dairy cattle as an indicator condition. Dispensing practices for other livestock diseases might differ, meaning the results cannot be assumed to represent antibiotic use across all veterinary conditions. The study documented the prevalence and patterns of non-prescription antibiotic dispensing but did not explore in detail the economic, cultural, or behavioral factors that influence non-veterinary personnel and farmers to engage in these practices. Some aspects of dispensing behavior, such as advice on withdrawal periods, may have been underreported or inconsistently provided by attendants due to the short interaction time with the simulated farmer.

The study focused on registered pharmacies and agro-veterinary shops. Informal and unregistered vendors, who may contribute significantly to inappropriate antibiotic use, were not included. Although simulated farmers were trained, recording information after leaving the outlets may have introduced recall bias or omission of details, particularly in busy dispensing interactions.

### 4.2 Strengths of the study

The study employed a robust and objective data collection approach that minimized social desirability and reporting bias. By using simulated farmers, it captured actual prescribing and dispensing practices rather than relying on self-reported behaviors. This is among the first systematic investigations of prescribing and dispensing practices of veterinary medicines for mastitis in dairy cattle by non-veterinary personnel in Zambia, generating new evidence to inform policy and stewardship interventions.

By examining inappropriate antibiotic use in food-producing animals, the study contributes directly to Zambia’s national and global efforts to mitigate AMR, a critical One Health challenge. The inclusion of six districts with significant dairy activity enhances the representativeness of the findings and allows for comparison across both rural and peri-urban settings.

Mastitis was strategically chosen due to its high prevalence, economic importance, and common reliance on antibiotics for treatment. This provided a practical lens to illustrate prescribing practices and potential misuse. The study not only assessed whether antibiotics were dispensed without prescriptions but also evaluated critical aspects of dispensing practices, including inquiry about symptoms, communication of dosage, duration, and referral practices. The findings offer actionable insights that can guide regulatory enforcement, AMS training, and farmer education in Zambia’s livestock sector, thereby strengthening the practical impact of the research.

### 4.3 Implications for policy and practice

This study highlights the deficiencies in dispensing practices of veterinary medicines by many non-veterinary personnel, particularly regarding the sale of veterinary antibiotics without a prescription. Such practices pose a significant risk for the misuse of antibiotics and the subsequent development of AMR in Zambia. Key findings revealed a high prevalence of non-prescription antibiotic dispensing by non-veterinary personnel, coupled with limited diagnostic inquiry and a lack of adherence to rational prescribing practices. To address these risks, targeted interventions are urgently needed. These include the implementation of AMS training programs for agro-veterinary and pharmacy personnel, public awareness campaigns for livestock owners, and the strengthening of enforcement mechanisms around prescription-only antibiotic sales. Collaborative engagement among dairy farmers, agro-veterinary shop owners, regulatory authorities, and veterinarians will be essential in promoting the responsible use of antimicrobials in the livestock sector.

However, several areas remain under-researched. There is limited understanding of the economic and behavioral drivers behind non-compliant prescribing and dispensing practices, especially in rural and informal market settings. Further investigation is also needed into the quality and supply chain dynamics of veterinary medicines in Zambia and neighboring countries. Moving forward, veterinary councils, pharmacy regulatory bodies, the ministry responsible for fisheries and livestock, ministry responsible for human health and One Health stakeholders should take the lead in developing integrated strategies to reduce inappropriate antimicrobial use and contain AMR across animal, human, and environmental health sectors.

The findings of this study underscore the urgent need for policy interventions to address the widespread non-prescription dispensing of veterinary antibiotics in Zambia. Strengthening enforcement of existing legislation such as the Veterinary and Para-Veterinary Professions Act and the Medicines and Allied Substances Act is critical to limit unauthorized antibiotic sales. Additionally, targeted AMS training for agro-veterinary and pharmacy personnel, coupled with public awareness campaigns for livestock farmers, can promote rational antibiotic use. Collaborative efforts between regulatory authorities, veterinary councils, and One Health stakeholders are essential to ensure sustainable policies that safeguard animal health, protect livelihoods, and mitigate the growing threat of AMR (Table 5).

**Table 5:** Policy implications, recommendations, and practice actions for AMR control in Zambia’s dairy sector.

Policy implications	Recommendations	Practice actions
Weak enforcement of prescription-only veterinary medicines has led to widespread non-prescription antibiotic dispensing by non-veterinary personnel.	Strengthen regulatory enforcement of the Veterinary and Para-Veterinary Professions Act (2010) and Medicines and Allied Substances Act (2013).	ZAMRA and Veterinary Council of Zambia to conduct routine inspections and enforce penalties for illegal dispensing. Introduce licensing tied to compliance with prescription-only policies.
High use of critically important antimicrobials (CIAs) in food-producing animals increases risk of resistance in both animals and humans.	Restrict and monitor use of WHO AWaRe “Watch” and “Reserve” group antimicrobials in veterinary practice.	Develop national guidelines for veterinary AMS including antibiotic categorization. Require veterinary prescription for all CIAs and high-priority antibiotics.
Limited diagnostic inquiry and irrational prescribing by non-veterinary personnel increases inappropriate antibiotic use.	Implement mandatory antimicrobial stewardship (AMS) training for agro-vet and pharmacy staff.	Train agrovet/pharmacy attendants on disease recognition, rational prescribing, and AMR risks. Introduce certification before being allowed to dispense antibiotics.
Poor communication on dosage, duration, and treatment monitoring undermines treatment outcomes and drives resistance.	Standardize treatment protocols for mastitis and other common livestock diseases.	Distribute simplified treatment guidelines in agrovet shops and pharmacies. Conduct refresher trainings and mentorship by veterinarians.
Low referral rates to veterinarians reduce professional oversight of animal treatment.	Encourage collaborative practice between non-veterinary personnel and veterinarians.	Create referral linkages where agrovet/pharmacy attendants must connect farmers to licensed veterinarians. Promote mobile veterinary services in underserved areas.
Limited awareness among farmers on risks of misuse of antibiotics.	Enhance public education campaigns on prudent antibiotic use and AMR risks.	Conduct farmer field schools, radio programs, and community awareness sessions. Encourage farmer cooperatives to adopt AMS practices.
Cross-cutting One Health concern: misuse of antibiotics in livestock threatens human health through food chain and environment.	Integrate veterinary AMS into Zambia’s National Action Plan on AMR (One Health approach).	Strengthen multi-sectoral collaboration between Ministry of Health, Ministry of Fisheries and Livestock, ZAMRA, and environmental agencies. Promote alternatives to antibiotics (vaccines, improved hygiene, biosecurity, and traditional remedies).

### 4.4 Recommendations of the study

#### 4.4.1 Policy and regulatory strengthening

To effectively address the widespread inappropriate use of veterinary antibiotics in Zambia, there is an urgent need to strengthen enforcement of existing legislation such as the Veterinary and Para-Veterinary Professions Act (2010) and the Medicines and Allied Substances Act (2013) to prevent unauthorized dispensing of antibiotics. In addition, the introduction of licensing and accreditation requirements for agro-veterinary shops and pharmacies should be linked to compliance with prescription-only antibiotic regulations. The use of CIAs and antibiotics classified under the “Watch” and “Reserve” groups should be strictly restricted and closely monitored through the development of targeted guidelines and the enforcement of formal prescription requirements. Furthermore, AMS should be integrated into Zambia’s National Action Plan on AMR, ensuring a coordinated One Health approach that connects animal, human, and environmental health to mitigate the growing threat of antimicrobial resistance.

#### 4.4.2 Practice and capacity building

To strengthen AMS and promote responsible antibiotic use in the livestock sector, it is essential to implement mandatory AMS training for agro-veterinary shop attendants and pharmacy staff to enhance their diagnostic inquiry, rational dispensing, and communication with farmers. In addition, simplified treatment protocols and guidelines for mastitis and other common livestock diseases should be developed and made readily available in agro-veterinary outlets and pharmacies to guide appropriate treatment decisions. Establishing formal referral systems that require non-veterinary personnel to connect farmers with licensed veterinarians for proper diagnosis and treatment would further improve the quality of care and reduce inappropriate antibiotic use. Moreover, farmer awareness should be enhanced through community-based education programs, farmer field schools, and media campaigns focusing on the risks of antibiotic misuse, the importance of observing withdrawal periods, and the broader implications of antimicrobial resistance. Finally, efforts should be made to promote alternatives to antibiotics, including the use of vaccines, improved farm hygiene, good milking practices, biosecurity measures, and proper nutrition, all of which can significantly reduce the reliance on antimicrobials in dairy production systems.

#### 4.4.3 Research and surveillance

There is a need to expand AMR and antimicrobial use surveillance in the dairy sector through systematic monitoring of prescribing practices, detection of antibiotic residues in milk, and assessment of resistance patterns in mastitis-causing pathogens. Further research should investigate the economic and behavioral drivers that influence non-veterinary personnel and farmers to engage in non-prescription antibiotic use, as understanding these factors is key to designing effective interventions. Additionally, pharmacovigilance systems should be strengthened to identify and eliminate substandard or counterfeit veterinary antibiotics from the supply chain, thereby ensuring the quality and efficacy of available medicines. Finally, operational research should be encouraged to evaluate the impact of AMS interventions, referral linkages, and alternative therapies on mastitis management and antibiotic use within Zambia’s dairy production systems.

## 5 Conclusion

This study provides critical insight into the prescribing and dispensing practices of veterinary medicines for the treatment of mastitis in dairy cattle by non-veterinary personnel in selected districts of Zambia. The findings reveal a high prevalence of non-prescription antibiotic dispensing, with limited diagnostic inquiry, poor communication on dosage and treatment duration, and frequent use of critically important antimicrobials for human medicine. These practices not only undermine effective treatment outcomes for mastitis but also accelerate the emergence and spread of AMR, threatening animal health, food safety, and public health within the One Health framework. The evidence underscores systemic weaknesses in regulatory enforcement, gaps in AMS knowledge, and insufficient collaboration between veterinary professionals and non-veterinary personnel. Left unaddressed, these challenges will continue to compromise animal productivity, increase the risk of antimicrobial residues in milk, and contribute to the growing global AMR crisis.

Addressing these issues requires a multipronged approach. Stronger enforcement of existing veterinary and pharmaceutical legislation is urgently needed to curb unauthorized dispensing. Mandatory AMS training for agro-veterinary and pharmacy personnel, alongside farmer education on prudent antibiotic use, can help improve dispensing practices and reduce inappropriate use. Collaborative frameworks that strengthen referral pathways between non-veterinary outlets and licensed veterinarians will also be key in ensuring professional oversight. Furthermore, integrating veterinary AMS into Zambia’s National Action Plan on AMR will help align interventions with broader One Health objectives. However, implementing these measures will require substantial financial, human, and infrastructural resources, as well as sustained political commitment. Challenges such as limited funding for regulatory enforcement, inadequate training capacity, and insufficient coordination among stakeholders may hinder progress unless adequately addressed.

In conclusion, the inappropriate prescribing and dispensing of veterinary antibiotics for mastitis by non-veterinary personnel present both a pressing veterinary and public health challenge. By strengthening regulation, improving training, and fostering intersectoral collaboration, Zambia has an opportunity to safeguard the effectiveness of antimicrobials, protect livelihoods in the dairy sector, and contribute to the global fight against AMR.

## Data Availability

The original contributions presented in the study are included in the article/Supplementary material, further inquiries can be directed to the corresponding author/s.
