# Supplementary material for: Non-prescription dispensing of veterinary medicines for treating mastitis in dairy cattle among non-veterinary personnel in selected districts of Zambia
Source: Front Vet Sci. 2026 Jan 2;12:1707434. doi: 10.3389/fvets.2025.1707434 (PMC12807936; doi:10.3389/fvets.2025.1707434)
Supplement: Supplementary file 1 [file Presentation_1.pdf]

Appendix I: Simulated farmer survey (Mystery Shopper Approach)

Serial No ________

A survey to evaluated prescribing practices of veterinary medicines in dairy cattle among non-veterinary personnel in Lusaka and Chongwe districts of Zambia

Please note that all the information will be treated as confidential

Participant's ID: ____________ Sex: _______

Name of interviewer: ___________

Section A: Dispensing Practice, Name and Type of Veterinary Medicine Dispensed

Gender of attendant:

a. Male
b. Female

Type of Premise visited

a. Retail Pharmacy
b. Agro-veterinary shop

Questions:

1) Did the attendant dispense any veterinary medicines without a prescription to you to treat your dairy cattle?
a. Yes
b. No

2) If yes, what name and type of veterinary medicine veterinary medicines did the attendant dispense to you
a. Name of antibiotic
b. Name of anthelmintic
c. Name of analgesic
d. Others (specify)

Section B: **Clinical symptoms, Condition, Previous Medicines Used, and Signs of infections to look out for in dairy cattle.**

Questions:

1) Did the attendant ask you about any possible symptoms of diseases in your dairy cattle?
a. Yes

b. No

2) Did the attendant tell you the possible diseases that dairy cattle present with?
a. Yes
b. No

3) Did the attendant ask about any previous medicines used in your dairy cattle?
a. Yes
b. No

4) Did the attendant tell you about any signs of infection to look out for in your dairy animals?
a. Yes
b. No

5) Did the attendant refer you to the veterinarian for specialized services?
a. Yes
b. No

Section C: Duration, Frequencies and Administration of Medicines Dispensed

Questions:

1. Did the attendant tell you about the frequency of administering medicines dispensed?
a. Not told
b. Once daily
c. Twice daily
d. Once every after 2 days (48:00 hrs)
e. Once every 3 days (72:00 hrs)
f. Do not stock
g. Other…………

2. Did the attendant tell you the duration of the treatment for the medicine dispensed?
a. Once off
b. 3 days
c. 3-5 days
d. 5 days
e. 7 days
f. Not told
g. Do not stock
h. Other…………

3. Did the attendant tell you how to administer the medicine dispensed?
a. Yes

b. No
c. Do not stock

4. If yes to question 3 above, how should the medicine be administered?
a. oral route (PO)

b. intramuscular (IM) route

c. Subcutaneous (SC) route

d. Intramammary route
